# Negro Doctors

**Published:** 1882-04-20

**Authors:** 


NEGRO DOCTORS.—Eight negroes were graduated at the Mehary Medical College of Nashville, Tenn.

SEE the new advertisement of Wm. R. Warner & Co., next to last page of reading matter.

